# Successful open radical gastrectomy for locally advanced or metastatic gastric cancer patients who suffered from coronavirus disease 2019 during preoperative chemotherapy: a report of three cases

**DOI:** 10.1186/s40792-022-01465-y

**Published:** 2022-06-24

**Authors:** Naoki Nishie, Manabu Ohashi, Rie Makuuchi, Masaru Hayami, Satoshi Ida, Koshi Kumagai, Souya Nunobe, Takeshi Sano

**Affiliations:** Department of Gastroenterological Surgery, Cancer Institute Hospital, Japanese Foundation for Cancer Research, 3-8-31 Ariake, Koto-ku, Tokyo, 135-8550 Japan

**Keywords:** COVID-19, Gastric cancer, Preoperative chemotherapy, Gastrectomy

## Abstract

**Background:**

According to previous reports, in patients with preoperative coronavirus disease 2019 (COVID-19) infection, mortality is increased if they undergo surgery within 6 weeks of diagnosis. However, the optimal timing and preoperative examination for gastrectomy with a previous COVID-19 infection are still controversial. We experienced three cases in which patients successfully underwent open radical gastrectomy following preoperative chemotherapy even though they developed COVID-19 infection during the chemotherapy.

**Case presentation:**

Case 1: A 58-year-old man with locally advanced gastric cancer caught COVID-19 during preoperative chemotherapy comprising 5-fluorouracil, calcium folate, oxaliplatin, and docetaxel. Although the patient had specific lung shadows indicating COVID-19 infection and deep venous thrombosis in the lower extremities, he underwent distal gastrectomy 10 weeks after the COVID-19 diagnosis. He had a good postoperative course. Case 2: A 56-year-old man with gastric cancer and lymph node and peritoneal metastasis caught COVID-19 during palliative chemotherapy comprising S-1, oxaliplatin, and trastuzumab. He underwent total gastrectomy as conversion surgery 8 weeks after COVID-19 infection. His postoperative course was uneventful. Case 3: A 55-year-old man with gastric cancer and paraaortic lymph node and liver metastases caught COVID-19 during S-1 and oxaliplatin treatment as neoadjuvant chemotherapy. He underwent distal gastrectomy, paraaortic lymph node sampling, and partial hepatectomy 8 weeks after COVID-19 infection although he had residual lung shadows and deep venous thrombosis in the lower extremities. He had an uneventful postoperative course.

**Conclusions:**

Computed tomography for preoperative evaluation was performed for all three patients and revealed that lung shadows remained post-COVID-19 infection. Despite this finding, the patients had good operative courses and were discharged as planned. Surgery after 7 weeks from the diagnosis of COVID-19 infection can be performed safely even when patients are post-chemotherapy and have residual lung findings and deep venous thrombosis. This report may contribute to the development of a consensus on performing safe gastrectomy for advanced gastric cancer in patients previously infected with COVID-19.

## Background

Coronavirus disease 2019 (COVID-19) has become a worldwide urgent public health concern. COVID-19 continues to spread around the world, with 400 million confirmed patients and more than 6 million deaths across almost 200 countries [1]. The pandemic has not yet been stamped out. In Japan, a state of emergency has been declared five times to date, and the total number of infected patients is estimated at > 5.6 million [2]. Although vaccination reduces the risk of catching and spreading COVID-19, the disease continues to spread. If this situation continues, there is concern that the number of patients with cancer who develop COVID-19 infection will increase in the near future.

One of the most problematic situations in cancer treatment associated with COVID-19 infection is when patients with resectable advanced cancer develop COVID-19 infection while they are waiting for surgery. Several concerns arise in this situation. The first concern is the timing of surgery. Recent reports recommend that operation be delayed because of the high risk of postoperative complications in patients with a history of COVID-19 infection [3]. However, a delay may lead to disease progression. Second, specific aftereffects of COVID-19 infection may influence a patient’s postoperative course. Possible impaired respiratory function and embolism formation can be associated with surgical morbidities. Third, considering the surgical risks, optimal or aggressive treatment may be cancelled. Additionally, modifications are required to decrease surgical radicality, which may reduce the potential for cure. COVID-19 is not only a respiratory infectious disease, but also a disease that can influence cancer prognosis.

In this report, we presented three cases of locally advanced or metastatic gastric cancer in unvaccinated patients against COVID-19, who caught COVID-19 during preoperative chemotherapy and who subsequently underwent successful open radical gastrectomy. Furthermore, we provided an extensive review of the literature and discussed how we should treat advanced gastric cancer patients with a history of COVID-19 infection shortly before planned surgery. In the future, surgeons will encounter many more patients who need treatment for both COVID-19 infection and resectable gastric cancer. This initial report may be a good reference for surgeons to help them decide how to treat patients with both diseases successfully.

## Case presentation

### Case 1

**Fig. 1:**
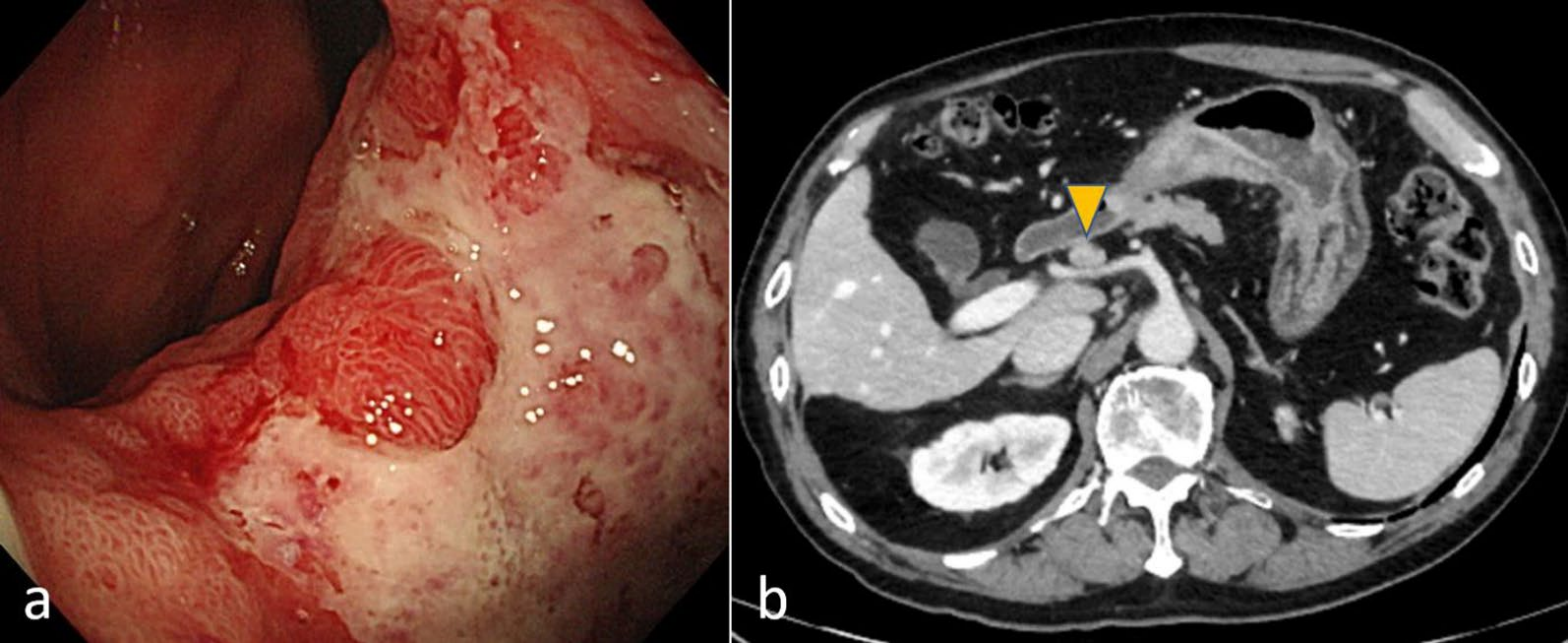
Findings of case 1 with gastrointestinal endoscopy and abdominal computed tomography before treatment. **a** A type 3 gastric cancer in the lesser curvature extending to the posterior wall of the middle to lower gastric body was found. **b** The station No. 8a lymph node (arrowhead) was enlarged

**Fig. 2:**
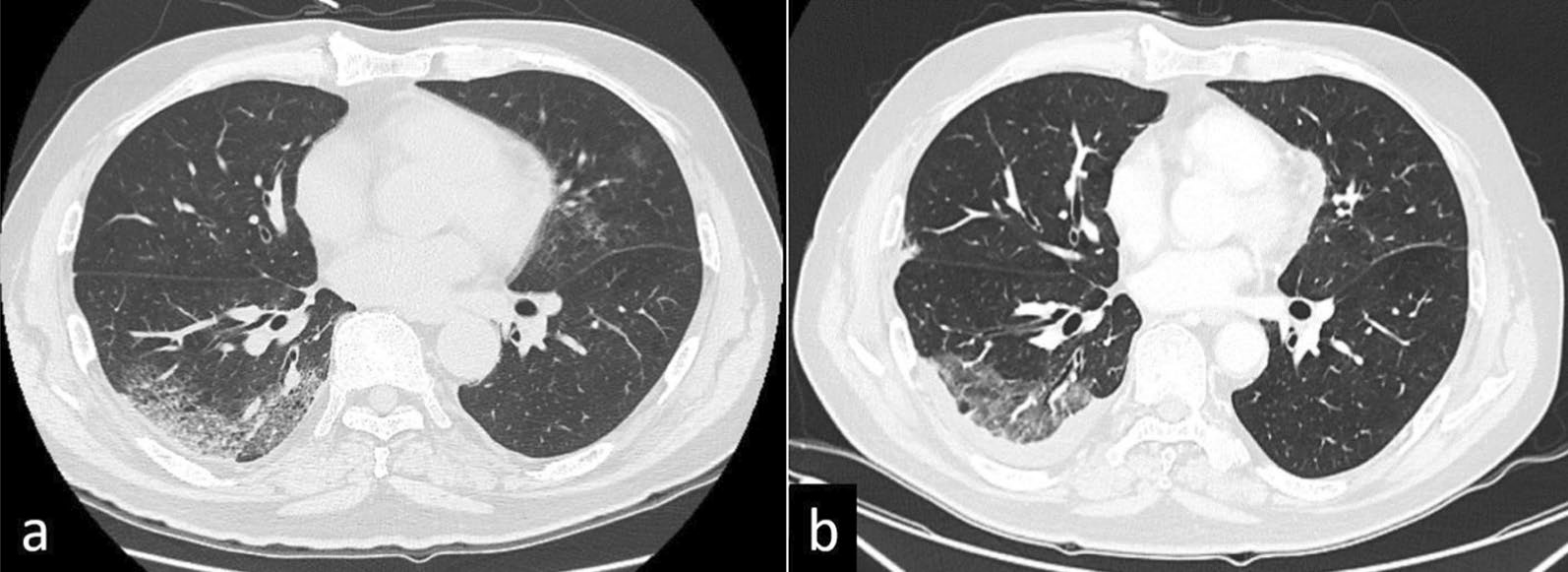
Lung findings of case 1 on chest computed tomography. **a** Peripheral ground-glass opacities and band-like opacities were found in bilateral lower lobes immediately after COVID-19 infection. **b** The ground-glass opacities regressed 23 days before the operation

A 58-year-old man who had visited a referral hospital owing to postprandial vomiting and dizziness underwent gastrointestinal endoscopy. A type 3 tumor measuring approximately 80 mm was identified in the lesser curvature of the middle to lower gastric body (Fig. 1a). Pathological findings in biopsy specimens revealed poorly differentiated adenocarcinoma and signet-ring cell carcinoma. Computed tomography (CT) detected lymph node enlargement around the common hepatic artery, but no distant metastases (Fig. 1b). The clinical diagnosis was stage III gastric cancer, and he was included in a clinical trial assessing preoperative combination chemotherapy using 5-fluorouracil, calcium folate, oxaliplatin, and docetaxel (FLOT) for resectable gastric cancer. He successfully finished two courses of FLOT as neoadjuvant treatment, which was behind schedule owing to adverse events. However, he developed a fever of 39 °C on the 22nd day after the start of chemotherapy. He urgently underwent a polymerase chain reaction (PCR) test and an antigen test for COVID-19 infection, with positive results for both tests. He was then hospitalized and observed in our facility. He had been almost asymptomatic on the initial day, but on the second day after admission, he complained of difficulty breathing owing to low blood oxygen saturation. Chest CT revealed a crazy-paving pattern, which can predict the severity of COVID-19, and a small number of ground-glass opacities (GGOs) located peripherally in bilateral lower lobes in addition to pleural effusion. These findings are typical of COVID-19 infection (Fig. 2a). He suffered from moderate pneumonia and was given remdesivir and corticosteroids. Follow-up CT revealed improvement of the pneumonia, and he was discharged on the 21st day of his hospital stay. On the 54th day after COVID-19 infection, chest–abdominal CT for preoperative evaluation revealed that the GGOs in the periphery of bilateral lungs remained, but that the pleural fluid had decreased in volume or had resolved (Fig. 2b). Preoperative pulmonary function testing revealed that the percentage vital capacity (%VC) was 102.4%, and the percentage of forced expiratory volume in 1 s (FEV1%) was 65.3%. Furthermore, blood testing showed elevated d-dimer levels, and ultrasonography identified the peripheral type of deep vein thrombosis (DVT) without free-floating thrombi in the lower extremities, for which he received apixaban. Subsequently, he underwent open distal gastrectomy with D2 lymph node dissection for ycStage III gastric cancer on the 73rd day after the COVID-19 diagnosis. The operation time was 290 min, and blood loss was 440 ml. To prevent perioperative pulmonary embolism, elastic stockings were used, and anticoagulant drugs were administered. The patient had a good postoperative course and was discharged on the 9th postoperative day. He is now undergoing adjuvant chemotherapy in accordance with the trial protocol.

### Case 2

**Fig. 3:**
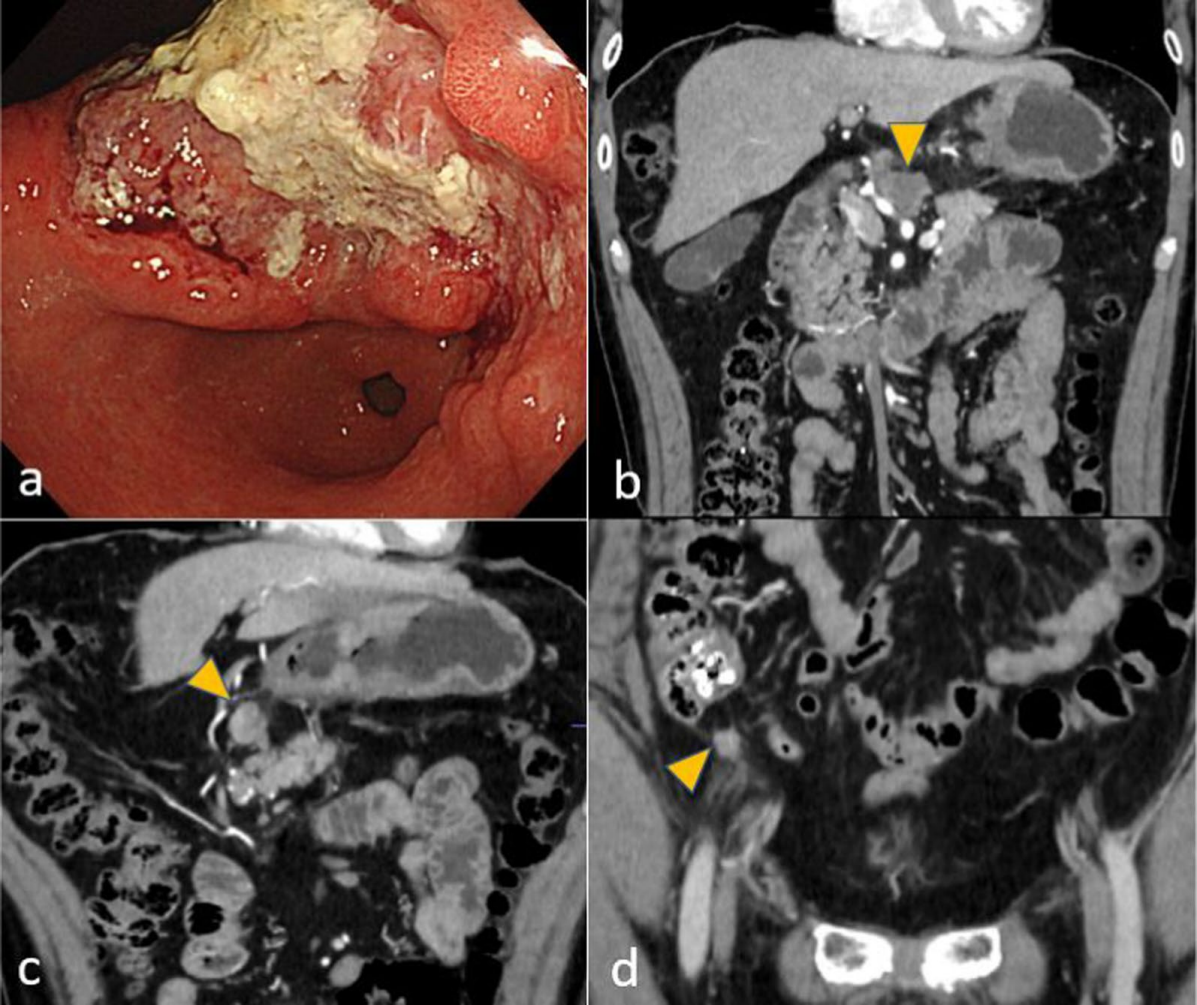
Findings of case 2 with gastrointestinal endoscopy and abdominal computed tomography before treatment. **a** A type 3 tumor in the lesser curvature of the upper to lower gastric body was found. **b**–**d** Enlarged station No. 6 and 8a lymph nodes and peritoneal metastasis near the cecum (arrowhead) were found

**Fig. 4:**
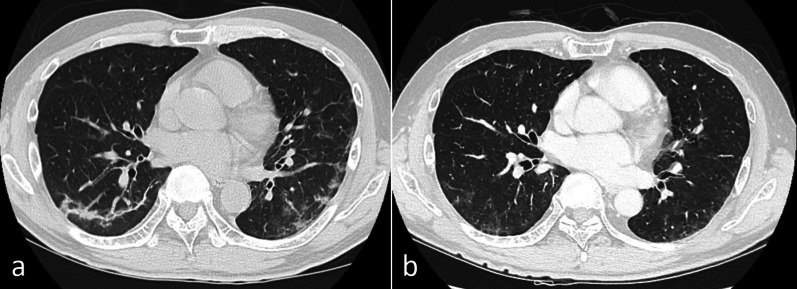
Findings of case 2 with chest computed tomography. **a** A crazy-paving pattern and small ground-glass opacities were located peripherally in bilateral lower lobes immediately after COVID-19 infection. **b** Ground-glass opacities were still present 19 days before the operation

A 56-year-old man who had suffered from a stomachache for half a year underwent an upper gastrointestinal series and gastrointestinal endoscopy. These procedures identified a type 3 gastric cancer measuring approximately 150 mm in the lesser curvature extending to the posterior wall of the upper to lower gastric body (Fig. 3a). The pathological findings of biopsy specimens revealed poorly differentiated adenocarcinoma and positive human epidermal growth factor receptor 2. Abdominal CT detected multiple enlarged lymph nodes and peritoneal metastases (Fig. 3b–d). Staging laparoscopy revealed gross peritoneal metastases but negative lavage cytology (P1CY0). He underwent combination chemotherapy comprising S-1 plus oxaliplatin (SOX) and trastuzumab as palliative treatment. In the ninth month after the start of chemotherapy, when he had undergone 17 courses of chemotherapy including some rest periods owing to his request, he suddenly developed a fever, with positive PCR test results for COVID-19 infection. Chest CT revealed typical GGOs and band-like opacities located peripherally in bilateral lower lobes (Fig. 4a). After hospitalization, he was considered a high-risk patient for COVID-19 pneumonia, and corticosteroids were administrated. He was discharged after 9 days of hospitalization. On the 36th day after the infection, chest–abdominal CT to evaluate his lung condition and the gastric cancer revealed that the abnormal lung findings had regressed compared with the previous images (Fig. 4b), and the enlarged abdominal lymph nodes had disappeared. Regarding pulmonary function, %VC and FEV1% were 98.2% and 78.4%, respectively. Other routine preoperative examinations identified no abnormal findings; thus, we considered removing the stomach as conversion surgery. On the 46th day after the infection, staging laparoscopy was performed again, which revealed no peritoneal metastasis. We then decided to perform total gastrectomy for ycT4aN0M0, ycStage IIB gastric cancer. On the 59th day, he underwent planned surgery successfully. The operation time was 290 min, and blood loss was 814 ml. To prevent pulmonary embolism, elastic stockings and intermittent pneumatic compression were used in addition to anticoagulant drugs. He had a good postoperative course and was discharged on the eighth postoperative day. He did not undergo adjuvant chemotherapy and is alive without relapse at the time of writing.

### Case 3

**Fig. 5:**
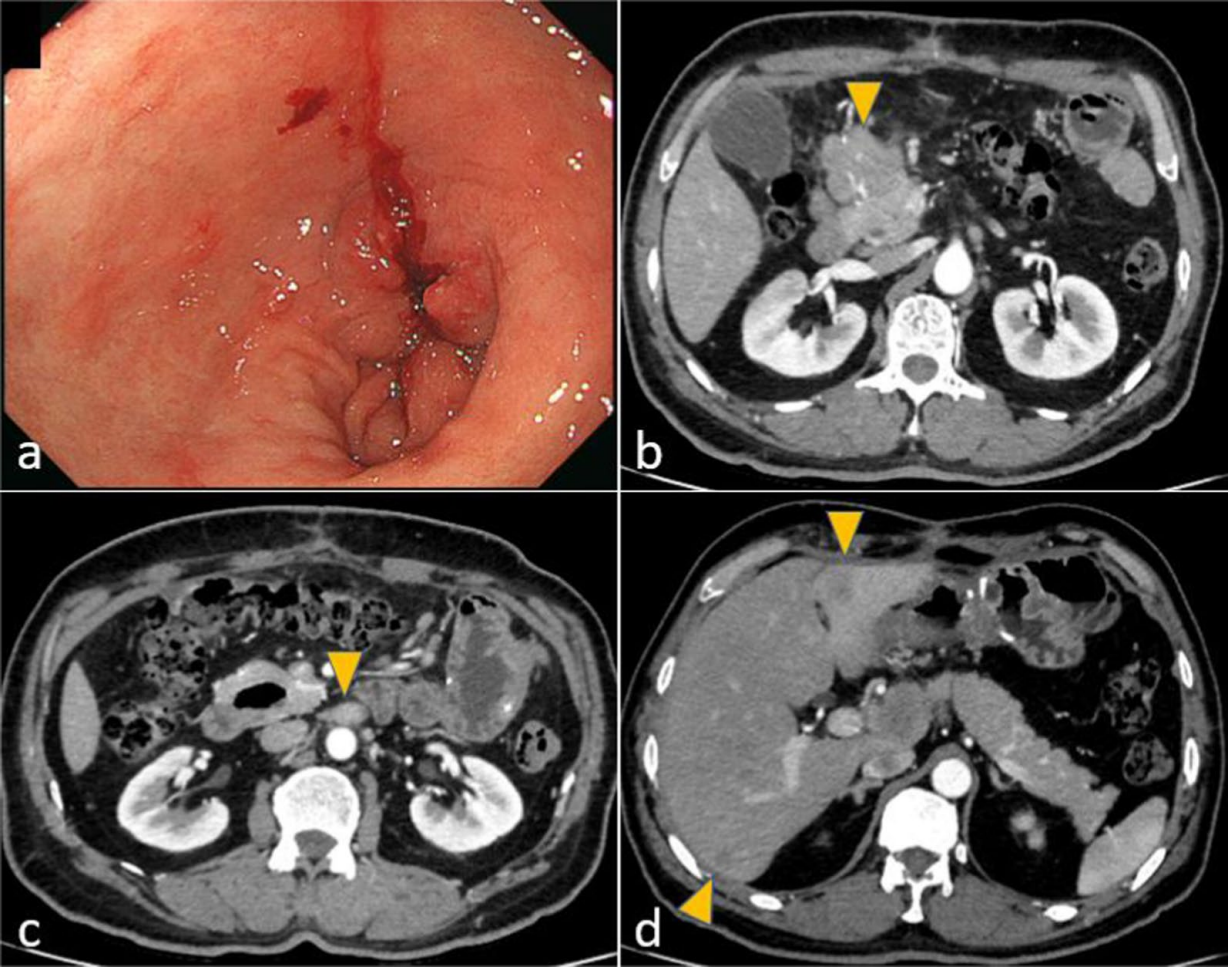
Findings of case 3 with gastrointestinal endoscopy and abdominal computed tomography. **a** A type 3 tumor caused circular stenosis from the antrum to the pylorus. **b**, **c** Station No. 6 and 16b1 lymph nodes were enlarged. The station No. 6 lymph node had invaded the pancreatic head. **d** Two small liver metastases were newly found during chemotherapy

**Fig. 6:**
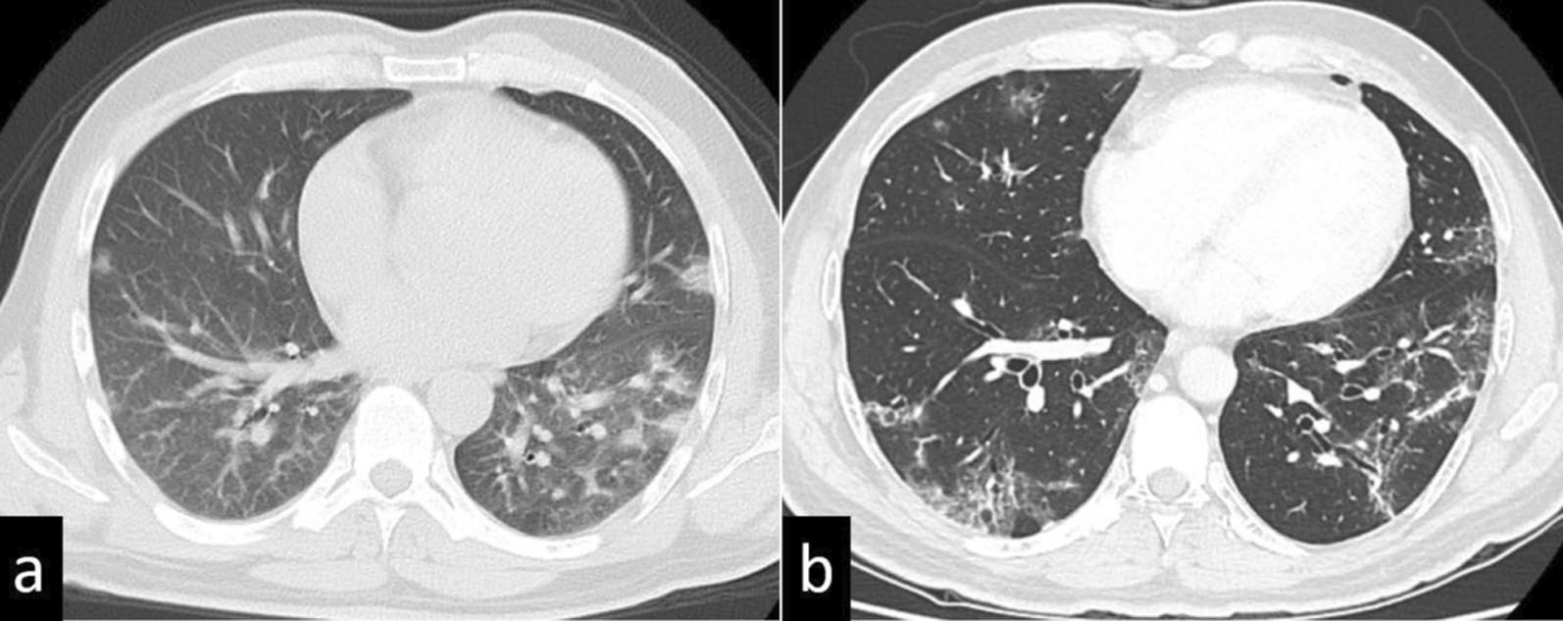
Findings of case 3 with chest computed tomography. **a** Ground-glass opacities and faint shadows were identified in bilateral lower lobes immediately after COVID-19 infection. **b** The opacities and shadows had regressed 19 days before the operation

A 55-year-old man visited a previous hospital, suffering from a stomachache. Gastrointestinal endoscopy identified a type 3 tumor measuring approximately 40 mm and circular stenosis from the antrum to the pylorus (Fig. 5a). Pathological evaluation of biopsy specimens revealed papillary adenocarcinoma. Abdominal CT detected multiple enlarged lymph nodes along the greater curvature and around the infrapyloric region and common hepatic arteries, as well as at the paraaortic area (Fig. 5b, c). He planned open distal gastrectomy; however, the tumor could not be removed owing to invasion of metastatic lymph nodes into the pancreatic head. He palliatively underwent gastrojejunostomy to be able to digest food. After discharge from the previous hospital, he was referred to our hospital with a request to resect the tumor. We intended to perform gastrectomy or pancreaticoduodenectomy following SOX therapy. During the treatment, abdominal CT for surveillance revealed two new small liver metastases, which were resectable (Fig. 5d). Seven courses of chemotherapy were administered; however, it was becoming more difficult to continue the treatment because of myelosuppression. Additionally, he developed a fever and had a positive PCR test result for COVID-19 infection. Chest CT identified GGOs and faint shadows located in bilateral lower lobes (Fig. 6a). Upon being diagnosed with moderate pneumonia, remdesivir and corticosteroids were administrated. Twenty-eight days of hospitalization were needed before discharging owing to another problem (bile duct stone). On the 42nd day after the infection, chest–abdominal CT revealed that the lung shadows had regressed compared with the previous images (Fig. 6b). Because the primary lesion, enlarged lymph nodes, and liver metastases had all decreased in size, we planned to perform surgery. The %VC and FEV1% values were 98.2% and 78.4%, respectively. The d-dimer concentration was elevated, and ultrasonography identified DVT in the lower extremities, for which apixaban was used. On the 61st day after the infection, planned open distal gastrectomy with D2 plus sampling of paraaortic lymph nodes, cholecystectomy, and partial liver resection (segments 3 and 6) was performed for ycT4aN0M1 (HEP), ycStage IVB gastric cancer. The operation time was 526 min, and blood loss was 490 ml, Anticoagulant drugs were used to prevent pulmonary embolism, postoperatively. He had a good postoperative course and was discharged on the ninth postoperative day. He is undergoing adjuvant chemotherapy at the time of writing.

## Discussion

In this report, we presented three cases of locally advanced or metastatic gastric cancer in patients who underwent radical gastrectomy following intensive chemotherapy despite developing a COVID-19 infection just before the surgery (Table 1). Although these were high-risk patients regarding COVID-19 infection because they had advanced gastric cancer and were receiving chemotherapy, the patients recovered smoothly from their COVID-19 infections and underwent planned curative gastrectomy. Even as the scope of the COVID-19 pandemic decreases, the number of gastric cancer patients with a history of COVID-19 infection will increase. In this case report, the treatment for gastric cancer was delayed to some extent but was not reduced in scope or aggressiveness, even though the patients had residual lung shadows and DVT. These initial cases will encourage surgeons to address the challenge of possible eradication of advanced cancer in patients with a history of COVID-19 infection.

**Table 1:** Summary of the patients’ characteristics in the three cases

Case	Age/sex	Preop CH	OP	IIS (days)	PN	Preop CT	%VC (pre-/post-infection: %)	FEV1% (pre-/post-infection: %)	DVT	Postop stay (days)
1	58/M	FLOT	ODG	73	Md	GGOs	120.2/102.4	66.8/65.3	+	9
2	56/M	SOX + HER	OTG	59	Md	GGOs	100.3/90.5	91.6/87.3	−	7
3	55/M	SOX	ODG + PH	61	Md	GGOs Faint shadows	116.8/98.2	77.3/78.4	+	8

*Preop* preoperative, *CH* chemotherapy, *CT* computed tomography, *%VC* percentage vital capacity, *Fev1%* forced expiratory volume in 1 s, *DVT* deep vein thrombosis, *Postop* postoperative, *M* male, *FLOT* 5-fluorouracil, calcium folate, oxaliplatin, and docetaxel, *SOX* S-1, oxaliplatin, *HER* herceptin, *OP* operative procedure, *ODG* open distal gastrectomy, *OTG* open total gastrectomy, *PH* partial hepatectomy, *IIS* interval between infection and surgery, *PN* pneumonia, *Md* moderate pneumonia, *GGOs* ground-glass opacities

Regarding the timing of surgery following COVID-19 infection, a previous report indicated that in patients with a preoperative COVID-19 diagnosis, the mortality rate increased if they underwent surgery within 6 weeks of the diagnosis [4, 5]. Mortality was twice as high as that of patients who underwent surgery ≥ 7 weeks after a COVID-19 diagnosis. Furthermore, after ≥ 7 weeks’ delay in undergoing surgery following COVID-19 infection, patients with ongoing symptoms had a higher mortality rate than that in patients whose symptoms had resolved or who were asymptomatic. Therefore, where possible, surgery should be delayed for at least 7 weeks following COVID-19 infection, and patients with ongoing symptoms for ≥ 7 weeks from diagnosis may benefit from a further delay. However, decisions should be tailored for each patient because the possible advantages of delaying surgery must be balanced against the potential risks associated with a delay. For some urgent surgical procedures, such as resection of advanced tumors, it may be decided that too long a delay in treatment is unjustified. All three cases presented in this report underwent gastrectomy > 7 weeks after their COVID-19 diagnosis. The patients had neither respiratory nor other symptoms associated with COVID-19 infection although they still had abnormal lung findings on imaging. Because these patients had locally advanced or metastatic gastric cancer, delayed surgery might have been a critical issue. However, several courses of intensive chemotherapy with a neoadjuvant or palliative intent might have negated the disadvantages of such a delay because chemotherapy decreased the size of their tumors. Usually, we perform gastrectomy 3–4 weeks after the end of chemotherapy; thus, the essential delay in the presented cases was substantially shorter than 7 weeks. In our cases, we found that radical gastrectomy after waiting for the recommended period was safe even though the patients still had typical findings of COVID-19 infection in their lungs, and the intended surgery followed intensive chemotherapy.

Chest CT is used for the assessment of the severity of patients suffering from COVID-19 infection and to help determine the management plan. However, chest CT is associated with the risk of radiation exposure or the risk of spreading infection in the hospital. Previous studies reported the CT findings of acute/subacute COVID-19 patients [6]. The main CT feature of COVID-19 pneumonia is the presence of GGOs, typically with a peripheral and subpleural distribution. The involvement of multiple lobes, particularly the lower lobes, is reported in the majority of infected patients. These areas of GGO may be admixed with areas of focal consolidation and/or associated with superimposed intralobular reticulations, resulting in a crazy-paving pattern. Notably, there is no consensus about how to handle patients who have abnormal findings on chest CT after COVID-19 treatment. A recent study reported that the mean total CT score and the number of involved lung lobes gradually decreased from admission; that is, 61% of patients had resolution of CT abnormalities 3 months after symptom onset, and at 12 months, 75% had resolution [7]. Thus, 25% of patients had abnormal findings on chest CT 12 months after a COVID-19 diagnosis, and 13% presented with subpleural reticular/cystic lesions. Age ≥ 50 years, lymphopenia, and aggravation of severe acute respiratory distress syndrome were independent risk factors for residual CT abnormalities at 1 year [7]. Severe COVID-19 pneumonia can lead to interstitial pneumonitis or pulmonary fibrosis, which may decrease respiratory function [7]. In the presented three cases, while preoperative CT revealed abnormalities, such as GGOs, and respiratory function declined slightly compared with pre-infection, the patients had no perioperative respiratory complications. When preoperative CT shows residual abnormal findings, we might not have to pay special attention to perioperative respiratory management if patients are asymptomatic or if respiratory function is maintained. However, ensuring that respiratory function is maintained 3 months after COVID-19 diagnosis is likely to be more acceptable when there is no urgency like surgery for early-stage cancer.

Some studies reported that COVID-19 is associated with an increased risk of developing venous thromboembolism (VTE) [8–10]. These studies indicated that the incidence of VTE in patients hospitalized for COVID-19 pneumonia was 4.4%, and 8.3–31% in patients with severe symptoms. In Japan, it has been thought that cancer, respiratory disease, and serious infection are high-risk conditions for VTE. Therefore, we used elastic stockings and intermittent pneumatic compression postoperatively in the patients in this report. Recent studies reported that d-dimer measurement for DVT may be superior to ultrasonography [11, 12]. However, elevated D-dimer can also be associated with malignant tumors, which may lead to excessive unnecessary use of anticoagulants. When high d-dimer is found in preoperative blood testing, we perform lower limb venous ultrasonography to detect DVT. If d-dimer is elevated in patients with previous COVID-19 infection, ultrasonography to detect DVT should definitely be performed, regardless of the d-dimer concentration, and drugs such as heparin can be administered in addition to the usual perioperative anticoagulant therapies, as appropriate. Of the three presented patients, high d-dimer concentration and DVT were found after COVID-19 infection in two patients. Although anticoagulant drugs were administered perioperatively in both patients, blood transfusion was not required, and there were no complications associated with bleeding.

## Conclusions

Because almost 2 years have passed since the beginning of the COVID-19 pandemic, strong evidence regarding the management of patients with previous COVID-19 infection is urgently required. On the basis of our findings for the three cases presented in this report, we conclude that patients with advanced gastric cancer and a recent history of COVID-19 infection can safely undergo radical gastrectomy at least 7 weeks from infection even with preoperative chemotherapy, asymptomatic residual lung shadows, and DVT. This report may contribute to the development of a consensus on performing safe gastrectomy for advanced gastric cancer in patients previously infected with COVID-19. Further research is needed to create risk stratification systems for surgery for patients with resectable gastric cancer and previous infection.

## Data Availability

Data sharing is not applicable to this article as no datasets were generated or analyzed during the current study.

## Abbreviations

COVID-19: Coronavirus disease 2019
CT: Computed tomography
FLOT: 5-Fluorouracil, calcium folate, oxaliplatin, and docetaxel
PCR: Polymerase chain reaction
GGO: Ground-glass opacity
%VC: Percentage vital capacity
FEV1%: Percentage of forced expiratory volume in 1 second
DVT: Deep vein thrombosis
SOX: S-1 and oxaliplatin
VTE: Venous thromboembolism
